# Lipid Nanoparticles Loaded with Iridoid Glycosides: Development and Optimization Using Experimental Factorial Design

**DOI:** 10.3390/molecules26113161

**Published:** 2021-05-25

**Authors:** Marta Dąbrowska, Eliana B. Souto, Izabela Nowak

**Affiliations:** 1 Department of Applied Chemistry, Faculty of Chemistry, Adam Mickiewicz University in Poznań, Uniwersytetu Poznańskiego 8, 61-614 Poznan, Poland; marta.dabrowska@amu.edu.pl; 2 Department of Pharmaceutical Technology, Faculty of Pharmacy, University of Coimbra, Polo das Ciências da Saúde, Azinhaga de Santa Comba, 3000-548 Coimbra, Portugal; souto.eliana@gmail.com; 3 CEB—Centre of Biological Engineering, Campus de Gualtar, University of Minho, 4710-057 Braga, Portugal

**Keywords:** lipid nanoparticles, iridoid glycosides, aucubin, catalpol, factorial design

## Abstract

Lipid nanoparticles based on multiple emulsion (W/O/W) systems are suitable for incorporating hydrophilic active substances, including iridoid glycosides. This study involved optimization of composition of lipid nanoparticles, incorporation of active compounds (aucubin and catalpol), evaluation of stability of the resulting nanocarriers, and characterization of their lipid matrix. Based on 3^2^ factorial design, an optimized dispersion of lipid nanoparticles (solid lipid:surfactant—4.5:1.0 wt.%) was developed, predisposed for the incorporation of iridoid glycosides by emulsification-sonication method. The encapsulation efficiency of the active substances was determined at nearly 90% (aucubin) and 77% (catalpol). Regarding the stability study, room temperature was found to be the most suitable for maintaining the expected physicochemical parameter values (particle size < 100 nm; polydispersity index < 0.3; zeta potential > |± 30 mV|). Characterization of the lipid matrix confirmed the nanometer size range of the resulting carriers (below 100 nm), as well as the presence of the lipid in the stable β’ form.

## 1. Introduction

The choice of the method of the synthesis of lipid nanoparticles is determined by the nature of the active substance to be incorporated, the target structure and size of the carrier, and the expected release profile of the encapsulated active ingredient, which is closely related to the intended application site [1,2]. The main problem associated with incorporating hydrophilic compounds into lipid nanoparticles is their risk of being expelled from the lipid core towards the external aqueous phase of the surfactant due to their high affinity for water. Consequently, the level of encapsulation efficiency achieved in dispersions of lipid nanoparticles containing substances of hydrophilic nature typically does not exceed 80% [3,4,5]. In this case, the recommended method is based on a multiple emulsion (W/O/W), which enables the incorporation of hydrophilic active substances and the formation of lipid nanoparticles with a size below 100 nm. In principle, the method consists in dissolving the active compound in the aqueous phase of the internal W/O emulsion formed as a result of intensive mixing and homogenization by means of a high-speed homogenizer. The resulting W/O emulsion is then dispersed in an aqueous surfactant solution by mechanical mixing to form a multiple W/O/W emulsion containing nanostructured carrier systems. The method requires high accuracy in terms of maintaining appropriate temperatures at subsequent process steps, as the dispersions of lipid nanoparticles obtained by applying this method display a trend for a relatively high polydispersity index [4,6].

**Figure 1:**
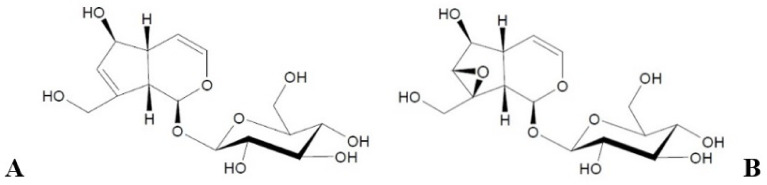
Structural formulas of aucubin (**A**) and catalpol (**B**).

Examples of hydrophilic active ingredients not previously reported in the literature to be loaded into lipid nanoparticles are iridoid glycosides, i.e., biologically active compounds of plant origin from the group of monoterpenoids [7,8,9,10,11]. Aucubin and catalpol (Figure 1) are among the most widespread iridoid glycosides in the plant world [7,9], and they are derived primarily from plants belonging to the family *Scrophulariaceae* [7,8]. With respect to their therapeutic effect, aucubin and catalpol have the ability to relieve swelling and produce an anti-exudative effect in a mechanism involving a decrease in the permeability of cutaneous blood vessels [12,13]. In addition, aucubin shows anti-inflammatory properties, inhibiting the activity of cyclooxygenase, which is responsible for the development of inflammation [13,14], while catalpol exerts a protective effect on liver cells [7,14]. Regarding their cosmetic properties, aucubin and catalpol enhance the regenerative processes of the epidermis during the induction of keratinocyte differentiation [15,16]. The combined effect of both substances improves the protective function of the lipid structures in the stratum corneum. By curbing inflammation, aucubin reduces age-related damage to the skin connective tissue because of the ability of the compound to stimulate the growth of fibroblasts [13,15,16]. In turn, catalpol plays a key role in processes associated with regulating an appropriate level of skin hydration [7,13,14]. At this point, it should be mentioned that iridoid glycosides have poor skin permeability as hydrophilic compounds. Nevertheless, these compounds to some extent penetrate through the epidermal barrier, leading to some beneficial effects related to the wound healing activity of aucubin [17] and therapeutic properties of catalpol in psoriasis treatments [18]. The potential of iridoid glycosides as cosmetic raw materials with anti-aging properties and a highly regenerative effect on the stratum corneum barrier could be combined with the beneficial cosmetic properties of lipid nanoparticles themselves. Due to their structure, based on lipophilic components, lipid nanoparticles exhibit adhesive and occlusive properties [5]. They form a protective film on the skin surface, limiting transepidermal water loss and maintaining an appropriate level of epidermal hydration [2,4,5]. Used in cosmetic formulations as carriers of active substances, they contribute to improving their penetration into the skin [2,4] and are expected to intensify the cosmetic action of aucubin and catalpol.

The aim of the study was to produce lipid nanoparticles loaded with selected iridoid glycosides (aucubin and catalpol) by applying the method of emulsification–sonication based on a multiple emulsion (W/O/W) and to optimize the quantitative composition of the resulting nanocarriers by applying the 3^2^ factorial design. In addition, the reported study involved the characterization of the physicochemical parameters of the obtained lipid nanoparticles and the lipid matrix, determination of the encapsulation efficiency, and evaluation of the stability of the obtained lipid nanoparticle dispersions by the techniques of dynamic light scattering (DLS), electrophoretic light scattering (ELS), transmission electron microscopy (TEM), differential scanning calorimetry (DSC), and X-ray diffraction (XRD). Finally, the applied research techniques sought to verify the potential for using optimized lipid nanoparticles incorporated with selected iridoid glycosides as a cosmetic raw material serving as an effective carrier system for active substances in cosmetic formulations.

## 2. Results and Discussion

### 2.1. Optimization of Quantitative Composition of Studied Lipid Nanoparticles (3^2^ Factorial Design)

Optimizing the composition of the lipid nanoparticle dispersions using statistical software is a way to develop a suitable lipid nanoparticle formulation with little expenditure of laboratory resources and at minimal costs while maximizing of the amount of data obtained [19,20,21]. The scheduled experiment (3^2^ factorial design) was aimed at obtaining stable dispersions of lipid nanoparticles suitable for the incorporation of the iridoid glycosides under study (aucubin and catalpol). Statistical analysis conducted using the 3^2^ factorial design [22,23] involved evaluating the impact of certain independent variables (solid lipid and nonionic surfactant content) on the dependent variables representing the basic physicochemical parameters of lipid nanoparticles (Z-ave: mean particle size, PDI: polydispersity index, ZP: zeta potential). The measured data obtained by determining the values of the dependent variables for all prepared samples of the lipid nanoparticle dispersions are listed in Table 1 and then analyzed statistically with the help of Statistica 10.0 software.

**Figure 2:**
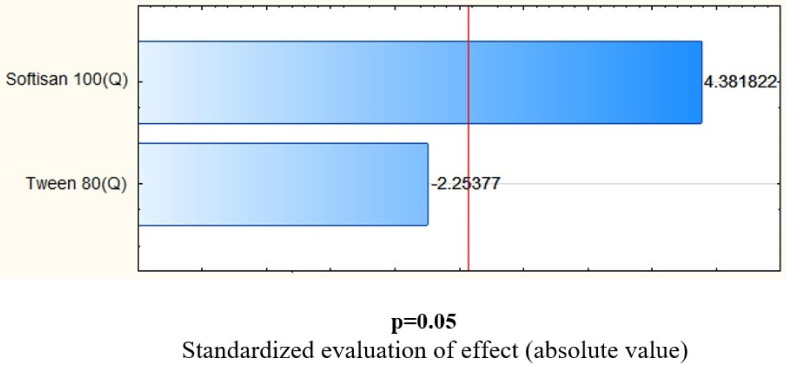
Pareto chart showing the effect of the lipid (Softisan^®^ 100) on mean particle size. The statistical analysis showed that average particle size of the prepared dispersions was dependent only on the percentage content of the solid lipid (*p* < 0.05). The impact of the amount of the surfactant (Tween^®^ 80) has been determined to be statistically insignificant.

**Figure 3:**
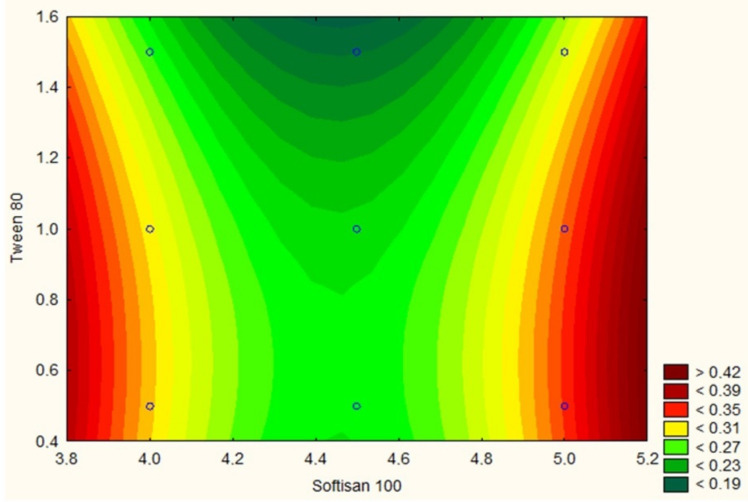
Response surface plot for the effect of the lipid (Softisan^®^100) and surfactant (Tween^®^ 80) content on polydispersity index. The determined level of polydispersity index was highly associated with the percentage content of Softisan^®^ 100 and Tween^®^ 80 (*p* < 0.05). Regarding the results, the optimum composition of the designed lipid nanoparticle dispersion was determined at the lipid to surfactant content ratio of 4.5:1.0 wt%.

The mean particle size determined for all obtained dispersions varied between 91.39 ± 1.97 nm and 147.10 ± 2.44 nm, while the polydispersity index values were shown to range from 0.214 ± 0.004 to 0.402 ± 0.009. The determined level of the two dependent variables was highly associated with the percentage content of the solid lipid—Softisan^®^ 100 for mean particle size (Figure 2), and additionally with the content of the nonionic surfactant—Tween^®^ 80 for the polydispersity index (Figure 3) of the prepared dispersions (*p* < 0.05). The zeta potential value varied from 9.09 ± 2.15 to 43.03 ± 2.06 mV and was found to be linked exclusively to the surfactant concentration (*p* < 0.05). In addition, it was determined that high positive results of zeta potential were associated with the presence of CTAB (hexadecyltrimethylammonium bromide)—a surfactant of cationic nature. The optimum composition of the designed lipid nanoparticle dispersion capable of incorporating iridoid glycosides was determined at the solid lipid to nonionic surfactant content ratio of 4.5:1.0 wt%. The physicochemical parameters of the optimized formulation of lipid nanoparticles were as follows: (i) Z-Ave = 93.32 ± 0.32 nm; (ii) PDI = 0.226 ± 0.004; (iii) ZP = 39.30 ± 4.22 mV. The selected dispersion was characterized by physicochemical parameters suitable for lipid nanoparticles intended for application as ingredients in cosmetic formulations. More precisely, a particle size below 100 nm is required for lipid nanoparticles intended for transdermal delivery [24]. Due to the fact that stratum corneum is a negatively charged membrane, positively charged lipid nanoparticles could remarkably enhance the penetration of active substances across the skin, attributed to the beneficial electrostatic interaction between the negatively charged skin surface and positively charged particles [25].

### 2.2. Assessment of Encapsulation Efficiency and Loading Capacity of Iridoid Glycosides

Encapsulation efficiency (EE) and loading capacity (LC) are the factors determining the correct selection of the qualitative and quantitative composition, and the method of obtaining lipid nanoparticle dispersions [4,26]. For each designed formulation of lipid nanoparticles, it is desirable to obtain the highest EE and LC possible, since their values determine the manner of release of the active substance from the lipid matrix (see the Supplementary Materials concerning the release study—Figure S1: Efficiency of release of iridoid glycosides from the cosmetic preparations) and the target efficacy of the obtained dispersions of lipid nanoparticles containing the active compounds under study [27,28,29]. The encapsulation efficiency and loading capacity of iridoid glycosides in lipid nanoparticles was determined using the technique of high-performance liquid chromatography. The amount of non-incorporated iridoid glycosides was calculated from the aucubin and catalpol concentrations determined in samples of the external aqueous phase of the surfactant based on the linear regression equations of previously generated calibration curves, which were then converted into the encapsulation efficiency and loading capacity (expressed as a percentage) of iridoid glycosides into the particles. The calculation was based on the theoretical assumption that for hydrophilic active compounds the EE value is at least 50% [4], so the total concentration of the non-incorporated iridoid glycoside in the test sample may not exceed 50 and 45 μg/mL for aucubin and catalpol, respectively. The results obtained by evaluating the efficiency of encapsulation and loading capacity of the iridoid glycosides in the lipid matrix are shown in Table 2.

The encapsulation efficiency of aucubin (89.52%) and catalpol (77.02%) as well as loading capacity—19.89 and 15.41%, respectively, were assessed as better than satisfactory, considering the nature of the incorporated iridoid glycosides and their low affinity towards the lipid nanoparticle matrix. The fact that the obtained EE and LC values exceeded significantly those typically associated with hydrophilic compounds can be attributed to the way the lipid nanoparticles under study were produced. The method of synthesis was based on a multiple W/O/W emulsion in which the active substances were incorporated into the aqueous phase of the internal emulsion. In this way, it is possible to decrease their undesirable penetration into the outer water phase of the surfactant, which reduces the level of their encapsulation efficiency. Comparing the results obtained for different iridoid glycosides, the noticeably higher value of encapsulation efficiency recorded for aucubin was attributed to differences in the solubility of the incorporated active compounds in water, which reached 66 and 60 mg/mL for aucubin and catalpol, respectively.

### 2.3. Evaluation of Stability of Studied Lipid Nanoparticles

The stability of lipid nanoparticles intended for incorporation into cosmetic formulations is evaluated in order to determine whether the designed dispersion of lipid nanoparticles is sufficiently stable to be used as an ingredient of cosmetic products that might then be potentially classified as nanocosmetics [30,31,32]. The stability of the obtained dispersions of lipid nanoparticles was studied by evaluating changes in the mean particle size, polydispersity index, and zeta potential [33,34] determined for the dispersions under analysis, stored under three temperatures (4, 25, and 40 °C) for the required period of 30 days. The choice of elevated temperature (40 °C) has been made in view of further plans for accelerated ageing tests of cosmetic formulations containing the developed lipid nanoparticles. The stability assessment was conducted for the dispersions of lipid nanoparticles (non-incorporated and incorporated with the iridoid glycosides tested) optimized by means of statistical analysis based on the 3^2^ factorial design—a sample with a qualitative and quantitative composition defined as Softisan^®^ 100 (4.5 wt%) and Tween^®^ 80 (1.0 wt%). The results obtained for the dispersions under analysis are listed in Table 3.

An analysis of data obtained by evaluating the stability of the studied dispersions of lipid nanoparticles (non-loaded and loaded with iridoid glycosides) showed that with respect to stability, the most favorable physicochemical parameters in terms of mean particle size and polydispersity index were obtained in the lipid nanoparticles stored at room temperature. Comparing changes in the values of these parameters over 30 days, determined for different temperature conditions, the following observations were made: (i) changes in the mean particle size by an average of 18.9, 3.7, and 9.3% (for the temperatures of 4, 25, and 40 °C, respectively, *p* < 0.05); (ii) changes in the polydispersity index by an average of 28.8, 2.0, and 3.6% (for the temperatures of 4, 25, and 40 °C, respectively, *p* < 0.05). No such correlation was found for the values of the zeta potential: their changes over time did not show a close relationship with the storage temperature of the samples. Moreover, the effect of incorporation of iridoid glycosides on the stability of the studied dispersions of lipid nanoparticles over time was determined in the form of decreases in the mean particle size and polydispersity index (on average by 23.4 and 19.9%, respectively, *p* < 0.05) and concurrent increases in the zeta potential, on average by 215.4% (the result was due to a decrease in the stability-lowering positive charge of the lipid nanoparticles under study, *p* < 0.05). It needs to be stated that iridoid glycosides by themselves do not carry a positive charge. However, due to their glycosidic moiety, the combination with other formulation components may cause such a noticeable enhancement of the values of zeta potential. The results obtained (mean particle size in the range of 79.30 ± 1.73–92.20 ± 3.85 nm; polydispersity index: 0.201 ± 0.013–0.297 ± 0.034; zeta potential: 34.37 ± 2.31–55.53 ± 1.55 mV) were found to be advantageous with respect to potential incorporation of the studied lipid nanoparticles into cosmetic formulations where, as previously mentioned, highly stable carrier structures with a size below 100 nm are desirable. A comparison of the results obtained for “empty” and “loaded” carrier systems shows that the lipid nanocarriers incorporated with iridoid glycosides are characterized by significantly smaller fluctuations in the values of the determined parameters over time. The reported correlation was particularly prominent when the dispersion was stored at reduced and elevated temperatures, when fluctuations in the mean particle size decreased by 74.9 and 98.5% (at 4 and 40 °C, respectively, *p* < 0.05), in the polydispersity index by 79.7 and 112.1% (at 4 and 40 °C, respectively, *p* < 0.05), and in the zeta potential by 53.8% (at 40 °C, *p* < 0.05) as a result of loading aucubin and catalpol into the lipid nanoparticles. The exception was the zeta potential determined for the samples stored at 4 °C. The value of this parameter rose significantly following the incorporation of the active substance (*p* < 0.05), while triggering significant fluctuations of the parameter during storage. The stability test results showed clearly that room temperature was the recommended level of storage temperature for the lipid nanoparticles loaded with aucubin and catalpol.

### 2.4. Analysis of Results of Characterization of Lipid Matrices

**Figure 4:**
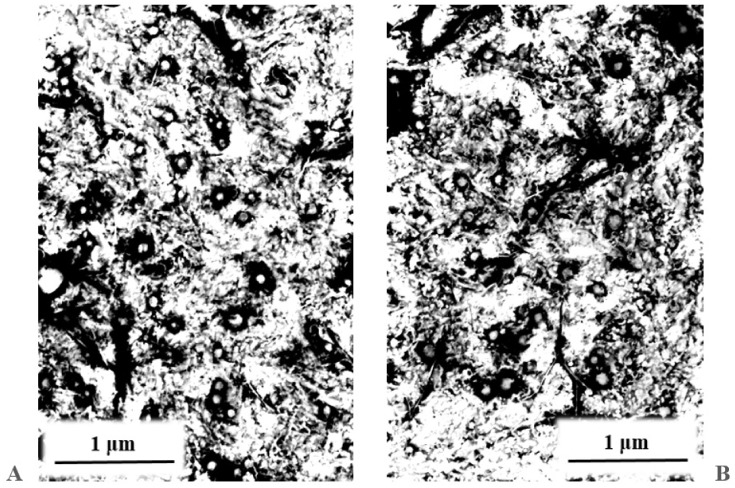
TEM images of lipid nanoparticles incorporated with iridoid glycosides tested proved the spherical shape of the obtained lipid nanoparticles and their size in the range below 100 nm. (**A**) Aucubin-loaded lipid nanoparticles with some polydisperse particles of size, which was still within the expected value range; (**B**) Catalpol-loaded lipid nanoparticles where no agglomeration was observed.

**Figure 5:**
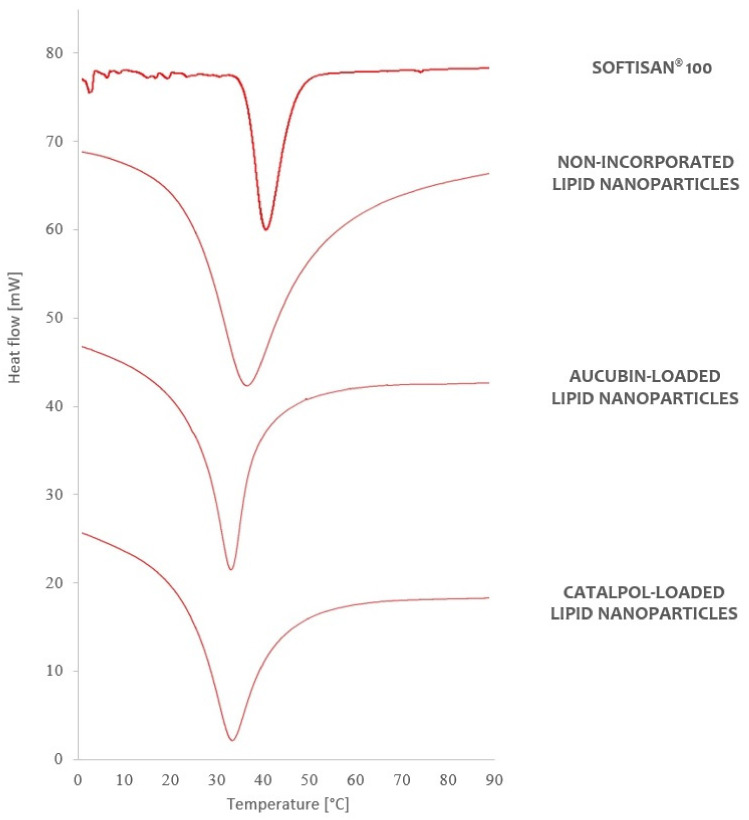
Comparison of DSC thermograms of the lipid and the studied lipid nanoparticle dispersions (the thermograms are shifted by a constant value of 20 mW in relation to the previous thermogram).

**Figure 6:**
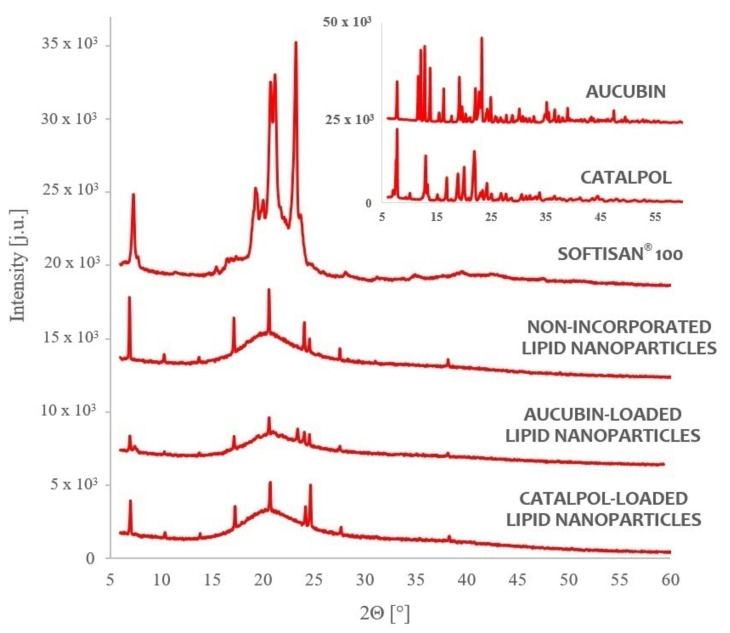
Comparison of diffractograms of the lipid and the studied dispersions of lipid nanoparticles (the diffractograms are shifted by a constant value of 6000 arbitrary units in relation to the previous diffractogram).

The characteristics of the lipid nanoparticles, and more specifically the lipid matrix constituting their fundamental element, have a bearing on the properties of the resulting carrier structures [27]. The physicochemical parameters of the lipid nanoparticle dispersions, in the form of efficiency of active substance encapsulation into lipid nanocarriers and the method of their release from the carrier, depend strictly on the morphological properties of the lipid used in the process of their synthesis. The lipid matrix of the obtained lipid nanoparticles was characterized by means of three compatible study techniques (TEM, DSC, XRD) that supplied data on the morphology, degree of crystallinity, and polymorphic variant of the lipid used [4,35,36]. The results of the characterization are shown in Figure 4 (TEM), Figure 5 (DSC), and Figure 6 (XRD).

#### 2.4.1. Transmission Electron Microscopy Analysis

Based on the images obtained by TEM, it was possible to assess the shape and morphology [37] of the studied dispersions of lipid nanoparticles loaded with iridoid glycosides. Figure 4 shows photographs of evaporated dispersions of the lipid nanoparticles loaded with aucubin (A) and catalpol (B). An analysis of TEM images provided evidence for the spherical shape of the obtained lipid nanoparticles and their size in the range below 100 nm, which predisposes them to be used as ingredients in cosmetic products. In the nanoparticles containing catalpol, no agglomeration was observed, while the image obtained for the aucubin-loaded structures showed polydisperse particles (with large statistical spread of particle sizes), the size of which was still within the expected value range (<100 nm). No crystals from the encapsulated active compounds were identified in the photographs. The TEM outcomes were shown to be consistent with the results of stability tests performed for the dispersions of lipid nanoparticles with the DLS method.

#### 2.4.2. Thermal Analysis

The next step in the characterization of the lipid matrix of the obtained nanoparticles was DSC analysis, for which it was assumed that the determination of the polymorphic variant of the lipid in the test sample would be based on the assumption that different polymorphic forms of the lipid (α, β’, β) have different melting points and corresponding enthalpy values [38,39]. The polymorphic nature of the lipid matrix was analyzed by comparing the DSC thermograms of the studied lipid nanoparticle dispersions with the DSC curve of the solid lipid (Figure 5).

Softisan^®^ 100, which is a mixture of hydrogenated triacylglycerols [2], in the solid state is present predominantly in the stable β form [4] appearing in the thermogram as a single endothermic peak at 42.73 °C, with enthalpy equal to −47.36 J/g. The conversion of the solid lipid to the colloidal state, associated with the formation of a lipid nanoparticle, caused a decrease in the melting point of the analyzed dispersion. For “empty” nanoparticles, a single endothermic peak was again observed at 38.96 °C, and higher enthalpy (−1.64 J/g) compared to the corresponding data obtained for the solid lipid. The observed changes in parameters were attributed to the partial transformation of the β form into the β’ form, which is characteristic of triacylglycerol mixtures in the colloidal state (the state in which the lipid occurs in the dispersion of lipid nanoparticles) [2,4,27]. The effect of active substance incorporation on further reduction of the melting point of the studied lipid nanoparticle dispersions was also confirmed. A characteristic endothermic peak was observed on the DSC curves of the carrier systems containing aucubin and catalpol, at reduced temperatures and increased enthalpy, demonstrating that the studied active compounds were successfully incorporated into the nanoparticles without disrupting the expected structure (β’ form) of the lipid matrix. The detailed values of the parameters determined by DSC analysis are listed in Supplementary Materials Table S1: DSC thermal analysis—parameters determined for the lipid and the studied dispersions of lipid nanoparticles.

#### 2.4.3. X-ray Studies

In order to fully characterize the lipid matrix, a XRD diffractogram analysis was performed to provide Supplementary Information on the degree of crystallinity and lipid polymorphism [40,41]. Figure 6 depicts the diffractograms recorded for the lipid and the studied dispersions of lipid nanoparticles, based on which they were compared with the solid lipid representing the reference sample. The crystallization profile of the solid lipid (Softisan^®^ 100) shows a characteristic signal at the angle 2Θ = 20.6°. The diffractograms of the studied dispersions of lipid nanoparticles showed a typical diffraction reflex at the angle 2Θ = 20.6°, confirming the presence of the polymorphic variant β’, characteristic of lipids based on triacylglycerols in the colloidal state [2,4]. Compared to the diffractogram of the solid lipid, a reduced intensity of diffraction of the identified signals (2Θ = 20.6°) was noted, amounting to (i) “empty” lipid nanocarriers—6304; (ii) nanocarriers incorporated with aucubin—3589; (iii) nanocarriers incorporated with catalpol—5137. These findings were consistent with the results obtained by DSC analysis, indicating the presence of the lipid in the β’ form in the nanoparticle matrix. Additionally, a higher degree of crystallinity was found in the dispersions of the nanoparticles incorporated with catalpol and aucubin compared to the non-incorporated lipid nanoparticles, based on compatibility determined between the enthalpy values (DSC analysis) and the intensity of diffraction signals (XRD analysis). Figure 6 also shows the diffractograms obtained for the pure active substances, aucubin and catalpol. However, the characteristic signals of such high intensity were no longer observed when analyzing the diffractograms recorded for the samples of lipid nanoparticles incorporated with the studied iridoid glycosides, which confirms the efficiency of loading of the formed lipid carriers during the synthesis by the method based on a multiple emulsion (W/O/W).

An analysis of characteristics of the lipid matrix of the obtained carrier systems confirmed that the applied method of producing lipid nanoparticles incorporated with iridoid glycosides was properly selected, enabling the synthesis of physically stable, spherical lipid nanoparticles of an appropriate size, showing no tendency to agglomeration (TEM). It was also confirmed that carrier structures based on a lipid in the β’ form were obtained, and the encapsulation of aucubin and catalpol into the nanoparticles was effective, not affecting the polymorphic form nor the stability of the lipid matrix (DSC, XRD).

## 3. Materials and Methods

### 3.1. Materials

Lipid nanoparticles were produced by using Softisan^®^ 100 (hydrogenated triacylglycerols as solid lipids) purchased from Sasol Germany GmbH (Hamburg, Germany), glycerol, CTAB (hexadecyltrimethylammonium bromide as a cationic surfactant), and Tween^®^ 80 (a mixture of polyethoxylated derivatives of sorbitan and oleic acid as a nonionic surfactant) from Sigma-Aldrich (Sintra, Portugal). Ultrapure distilled water was obtained using Milli-Q^®^ Plus system (Millipore, Germany). The incorporated active compounds belonging to the group of iridoid glycosides—aucubin and catalpol—were purchased from Angene International Limited (London, UK). The chromatographic analysis determining the efficiency of encapsulation was conducted using HPLC water (Avantor Performance Materials, Gliwice, Poland), trifluoroacetic acid (Sigma Aldrich, Poznan, Poland), and acetonitrile (Avantor Performance Materials, Gliwice, Poland).

### 3.2. Experimental Factorial Design

The quantitative composition of the lipid nanoparticle dispersions suitable for the incorporation of hydrophilic active substances—including selected iridoid glycosides (aucubin and catalpol)—was optimized by using the 3^2^ factorial design with the help of Statistica 10.0 software (StatSoft). The experiment required the selection of independent and dependent factors. Accordingly, two factors (each at three concentration levels) were adopted as independent variables: (i) solid lipid content: Softisan^®^ 100–4.0; 4.5; 5.0 wt%; (ii) nonionic surfactant content: Tween^®^ 80–0.5; 1.0; 1.5 wt%. Three parameters were selected as dependent variables: (i) mean particle size (Z-Ave), (ii) polydispersity index (PDI), and (iii) zeta potential (ZP). The 3^2^ factorial design required nine individual experiments (Table 4), prepared according to the procedure using a modified emulsification-sonication method based on a multiple emulsion (W/O/W) described precisely in Section 3.3. For each of the prepared samples, the values of the predefined dependent variables (Z-Ave, PDI, ZP) were measured in triplicate. The methodology of the measurements is provided in subsection “Physicochemical characterization” in the manuscript and in the Supplementary Materials. The statistical analysis involved an evaluation of measured data with the help of Statistica 10.0 software. The results of the 3^2^ factorial design were presented in the form of Pareto diagrams and 2D response surface plots. The level of statistical significance was set at *p* < 0.05.

### 3.3. Production of Lipid Nanoparticles

Lipid nanoparticle dispersions were produced by applying a modified emulsification-sonication method based on a multiple emulsion (W/O/W) [19]. The ingredients of the lipid phase were introduced to a glass beaker: (i) Softisan^®^ 100–4.0; 4.5; 5.0 wt%; (ii) glycerol—37.5 wt%; (iii) CTAB—0.5 wt%, and heated to 50 °C. Concurrently, a small amount of Milli-Q^®^ Plus water was added to another beaker and also heated to 50 °C. Subsequently, the aqueous phase thus prepared was combined with the lipid phase, upon vigorous stirring the emerging emulsion using a high-speed Ultra-Turrax^®^ T25 Digital Homogenizer (Ystral GmbH D-7801, Ballrechten-Dottingen, Germany) at a speed of 24,000 rpm for approximately 5 min. Next, the obtained W/O emulsion was homogenized using a VC130 Ultrasonic Processor (Sonics, Newtown, CT, USA) for 30 s at 40% amplitude. Concurrently, an aqueous solution of the surfactant Tween^®^ 80 (0.5; 1.0; 1.5 wt%) was prepared by dispersing it in Milli-Q^®^ Plus water and heating in order to obtain a homogeneous dispersion. In the next step, a small amount of the cooled aqueous surfactant solution was added to the previously prepared W/O emulsion. The resulting mixture was sheared (homogenized) using an ultrasonic homogenizer for 90 s at an amplitude of 40%. After that, the remainder of the aqueous surfactant solution was added to the obtained dispersion and stirred with a magnetic stirrer at 300–350 rpm until the cooling-down of the system (approx. 15 min). The procedure of incorporating aucubin and catalpol (each of the glycosides separately) into the lipid nanoparticles consisted in adding appropriate amounts of the active compounds (0.1 wt% of aucubin and 0.09 wt% of catalpol) to the aqueous phase of the inner W/O emulsion.

### 3.4. Physicochemical Characterization

Physicochemical parameters of the obtained dispersions of lipid nanoparticles—mean particle size, polydispersity index, and zeta potential—were determined with a Zetasizer Nano ZS (Malvern Instruments, Malvern, UK), using the techniques of dynamic light scattering [42] and electrophoretic light scattering [43] (see the Supplementary Materials for details).

### 3.5. Encapsulation Efficiency and Loading Capacity

The encapsulation efficiency (EE) and loading capacity (LC) of iridoid glycosides in the obtained lipid nanoparticles was determined using a high-performance liquid chromatograph (Varian 920-LC, Agilent Technologies, Santa Clara, CA, USA), coupled to a UV-Vis detector. The chromatographic determination of iridoid glycosides in the dispersion of lipid nanoparticles involved the quantification of non-incorporated active substance present in the external aqueous phase of the surfactant. The values obtained for the concentrations of the studied iridoid glycosides were converted into encapsulation efficiency and loading capacity (expressed as a percentage), in accordance with the following equations [41]:

(1) $$EE\;\left[\%\right]=\frac{total\;amount\;of\;active\;substance-amount\;of\;non-incorporated\;active\;substance}{total\;amount\;of\;active\;substance}$$

(2) $$LC\;\left[\%\right]=\frac{total\;amount\;of\;active\;substance-amount\;of\;non-incorporated\;active\;substance}{total\;amount\;of\;lipid}$$

The step-by-step procedure is provided in the Supplementary Materials.

### 3.6. Stability Test

The stability of the obtained dispersions of lipid nanoparticles was evaluated using a Zetasizer Nano ZS instrument and included the measuring of three characteristic physicochemical parameters: Z-Ave, PDI, and ZP. The stability test was performed at room temperature 24 h after the synthesis of the dispersions of lipid nanoparticles (day 1) and after 30 days (day 30) by analyzing samples stored under three temperature conditions (4, 25, and 40 °C).

### 3.7. Characterization of Lipid Matrices

#### 3.7.1. Transmission electron microscopy (TEM)

The morphology and shape of the obtained lipid nanoparticles were established on the basis of images recorded using a JEM-1200 EX II electron microscope (JEOL, Tokio, Japan). The test samples were prepared by drying the lipid nanoparticle dispersions at room temperature and performing negative staining with 2% uranyl acetate.

#### 3.7.2. Differential Scanning Calorimetry (DSC)

The polymorphic forms of the lipid matrix of the obtained lipid nanoparticles were evaluated on the basis of results of thermal analysis performed with a DSC 8500 differential scanning calorimeter (PerkinElmer, Waltham, MA, USA). For each sample, the procedure was performed once, and the results were presented in the form of DSC curves (graph showing the relationship between the measured difference in heat flow and temperature) and the determined melting point and enthalpy of the reaction (see the Supplementary Materials for the description of sample preparation).

#### 3.7.3. X-ray Diffraction (XRD)

The crystallinity and polymorphic forms of the lipid matrix of the obtained lipid nanoparticles were determined based on the results of diffraction analysis carried out with a D8 Advance powder diffractometer combined with a Johansson monochromator (Bruker, Billerica, MA, USA). To prepare the samples for analysis, the dispersions of lipid nanoparticles were placed on a measuring table and dried at room temperature. For each sample, the procedure was performed once, within the high-angle range (2Θ = 6.0–60.0°). The results were presented as diffractograms.

## 4. Conclusions

The therapeutic and cosmetic properties of chemical compounds from the group of iridoid glycosides—aucubin and catalpol—provided a foundation for a study involving an attempt to incorporate them into lipid nanoparticles designed for application as raw materials serving as effective carriers of active substances in cosmetic formulations. Optimization of the composition of the lipid nanoparticle dispersions is a step that precedes the actual process of their preparation based on a predefined, optimized formula. The essence of optimizing the composition of lipid nanoparticles is the possibility to produce dispersions that are suitable for the incorporation of the planned active compounds and stable under various temperature conditions for a specified period of time. This is especially relevant when designing the composition of lipid nanoparticles (i) containing active substances, the incorporation of which was not previously described in the literature (such as aucubin and catalpol investigated in this study); (ii) intended for the incorporation of active substances with non-lipophilic properties; and (iii) obtained with methods based on a multiple emulsion, sensitive to proper selection of synthesis conditions [19,20,21]. The reported studies made it possible to establish the optimum qualitative and quantitative composition of the dispersion of lipid nanoparticles—Softisan^®^ 100 (4.5 wt%) and Tween^®^ 80 (1.0% wt%)—suitable for the incorporation of iridoid glycosides by means of a modified emulsification and sonication method based on a multiple emulsion (W/O/W), which was selected due to the hydrophilic nature of the active compounds under study. Attempts were also made to incorporate aucubin and catalpol into the prepared lipid carriers, achieving the encapsulation efficiency of 89.52% (aucubin) and 77.02% (catalpol). The characterization of the lipid matrix performed in the next stage (confirming the spherical shape of the carrier structures and the stable β’ form of the lipid), as well as the evaluation of stability of the obtained dispersions (mean particle size—Z-Ave < 100 nm; polydispersity index—PDI < 0.3; zeta potential—ZP > |± 30 mV|) validated the optimal selection of the synthesis method and, at the same time, allowed the assessment of the resulting lipid nanoparticles as sufficiently stable to be applied as ingredients in cosmetic formulations.

## Figures and Tables

**Table 1:** Measured data obtained by conducting the 3^2^ factorial design (data are expressed as mean ± SD ^1^).

Sample	Mean Particle Size (nm, ±SD ^1^)	Polydispersity Index (-, ±SD ^1^)	Zeta Potential ^2^ (mV, ±SD ^1^)
A	99.46 ± 0.40	0.247 ± 0.013	9.09 ± 2.15
B	92.58 ± 0.81	0.276 ± 0.006	26.97 ± 6.06
C	91.39 ± 1.97	0.313 ± 0.027	16.00 ± 4.33
D	94.06 ± 0.61	0.214 ± 0.004	12.93 ± 1.87
E	93.32 ± 0.32	0.226 ± 0.004	39.30 ± 4.22
F	124.03 ± 2.32	0.370 ± 0.011	29.73 ± 2.61
G	116.63 ± 1.39	0.346 ± 0.008	17.57 ± 2.61
H	147.10 ± 2.44	0.402 ± 0.009	43.03 ± 2.06
I	112.07 ± 1.01	0.320 ± 0.007	39.23 ± 7.24

^1^ SD: standard deviation; ^2^ pH of solutions equal to approximately 6.2.

**Table 2:** Quantitative analysis of iridoid glycosides non-incorporated into the obtained lipid nanoparticles.

Incorporated Active Substance	Concentration (μg/mL ± SD ^1^)	EE ^2^ (% ± SD ^1^)	LC^2^ (% ± SD ^1^)
aucubin	10.476 ± 0.235	89.52 ± 0.72	19.89 ± 0.23
catalpol	20.683 ± 0.192	77.02 ± 0.93	15.41 + 0.46

^1^ SD: standard deviation; ^2^ calculated from the equations presented in subsection “Encapsulation efficiency and loading capacity”.

**Table 3:** Changes in mean particle size, polydispersity index, and zeta potential of the studied dispersions of lipid nanoparticles stored in various temperature conditions (4, 25, and 40 °C) for 30 days.

MEAN PARTICLE SIZE (nm, ±SD)				
		Blank ^1^	+aucubin	+catalpol
**temp. 4 °C**	**day 1**	100.97 ± 0.75	79.30 ± 1.73	82.55 ± 2.37
	**day 30**	139.10 ± 9.66	92.20 ± 3.85	84.78 ± 4.20
**temp. 25 °C**	**day 1**	93.32 ± 0.32	84.35 ± 1.84	84.79 ± 1.32
	**day 30**	103.93 ± 1.01	82.95 ± 2.65	85.86 ± 3.16
**temp. 40 °C**	**day 1**	139.57 ± 2.58	82.81 ± 1.56	81.84 ± 1.93
	**day 30**	101.73 ± 2.25	81.27 ± 2.31	82.66 ± 1.85
**POLYDISPERSITY INDEX (-, ±SD)**				
		Blank ^1^	+aucubin	+catalpol
**temp. 4 °C**	**day 1**	0.295 ± 0.021	0.236 ± 0.005	0.237 ± 0.003
	**day 30**	0.476 ± 0.085	0.297 ± 0.034	0.235 ± 0.003
**temp. 25 °C**	**day 1**	0.226 ± 0.004	0.240 ± 0.007	0.240 ± 0.014
	**day 30**	0.266 ± 0.018	0.232 ± 0.003	0.220 ± 0.009
**temp. 40 °C**	**day 1**	0.406 ± 0.010	0.237 ± 0.013	0.201 ± 0.013
	**day 30**	0.463 ± 0.021	0.216 ± 0.008	0.212 ± 0.013
**ZETA POTENTIAL (mV, ±SD)**				
		Blank ^1,2^	+aucubin ^3^	+catalpol ^3^
**temp. 4 °C**	**day 1**	9.37 ± 2.19	41.63 ± 6.44	50.77 ± 0.15
	**day 30**	8.82 ± 2.74	35.45 ± 1.91	41.60 ± 2.25
**temp. 25 °C**	**day 1**	39.30 ± 4.22	47.30 ± 1.47	55.53 ± 1.55
	**day 30**	15.50 ± 0.66	34.37 ± 2.31	37.07 ± 3.11
**temp. 40 °C**	**day 1**	15.30 ± 2.39	45.20 ± 0.95	53.43 ± 1.77
	**day 30**	13.17 ± 0.71	45.47 ± 1.10	46.23 ± 2.10

^1^ Blank: lipid nanoparticle before the incorporation of the active substance; ^2^ pH of solutions equal to approximately 6.2; ^3^ pH of solutions equal to approximately 6.0.

**Table 4:** Quantitative composition of samples of lipid nanoparticle dispersions prepared as part of the 3^2^ factorial design.

	Quantity of ingredient (wt% ± 0.01)				
Sample	Softisan^®^ 100 ^1^	Tween^®^ 80 ^2^	Glycerol	CTAB ^3^	Water Milli-Q^®^ Plus
A	4.00	0.50	37.50	0.50	56.00
B	4.00	1.00	37.50	0.50	55.50
C	4.00	1.50	37.50	0.50	55.00
D	4.50	0.50	37.50	0.50	55.50
E	4.50	1.00	37.50	0.50	55.00
F	4.50	1.50	37.50	0.50	54.50
G	5.00	0.50	37.50	0.50	55.00
H	5.00	1.00	37.50	0.50	54.50
I	5.00	1.50	37.50	0.50	54.00

^1^Softisan® 100 (solid lipid); ^2^ Tween^®^ 80 (nonionic surfactant); ^3^ CTAB (cationic surfactant).

## Data Availability

The data presented in this study are available upon request from the authors.

## Supplementary Materials

The following are available online. Figure S1: Efficiency of release of iridoid glycosides from the cosmetic preparations. Table S1: DSC thermal analysis—parameters determined for the lipid and the studied dispersions of lipid nanoparticles.

## Sample Availability

Samples of the catalpol and aucubin are commercially available, while emulsions can be prepared upon reasonable request.
