# Supplementary material for: Lipid Nanoparticles Loaded with Iridoid Glycosides: Development and Optimization Using Experimental Factorial Design
Source: Molecules. 2021 May 25;26(11):3161. doi: 10.3390/molecules26113161 (PMC8198468; doi:10.3390/molecules26113161)
Supplement: Supplementary file 1 [file molecules-26-03161-s001.zip › molecules-1164859-supplementary.pdf]

Supplementary Material

Lipid Nanoparticles Loaded with Iridoid Glycosides: Development and Optimization Using Experimental Factorial Design

Marta Dąbrowska [1,2], Eliana B. Souto [2,3] and Izabela Nowak [1,*]

1 Department of Applied Chemistry, Faculty of Chemistry, Adam Mickiewicz University, Poznań, Uniwersytetu Poznańskiego 8, 61-614 Poznan, Poland; marta.dabrowska@amu.edu.pl
2 Department of Pharmaceutical Technology, Faculty of Pharmacy, University of Coimbra, Polo das Ciências da Saúde, Azinhaga de Santa Comba, 3000-548 Coimbra, Portugal; souto.eliana@gmail.com
3 CEB—Centre of Biological Engineering, Campus de Gualtar, University of Minho, 4710-057 Braga, Portugal
* Correspondence: nowakiza@amu.edu.pl; Tel.: +48-61-829-1580

1. Physicochemical Characterization

The measurement was preceded by the preparation of aqueous solutions of the studied lipid nanoparticles (100 µL of the dispersion in 25 mL of distilled water), and determination of the refractive index in each test sample using a Refracto 30 PX/GS refractometer (Mettler Toledo, Warsaw, Poland). To perform the measurement, a 1 mL portion of the prepared sample was transferred by means of a syringe to the measuring cell which was then placed in the measuring chamber of the device. The test was carried out at room temperature. The procedure was performed in triplicate for each test sample, and the arithmetic mean and standard deviation were determined from the measurement results.

2. Encapsulation Efficiency and Loading Capacity

The samples were prepared by measuring 1 mL of the studied dispersion of lipid nanoparticles into an Eppendorf Tubes® test tube, placing it in the angular rotor of a MPW-350R laboratory centrifuge, and centrifuging at 3,000 rpm for 30 min. In the next step, a 9 mL portion of distilled water was added to the aqueous solution of the external phase separated by centrifugation, and the sample was shaken vigorously for approx. 5 min. The resulting solution was passed through a syringe filter with a pore size of 0.45 µm, and a 1.5 mL portion was transferred to a glass vial for HPLC analysis. The chromatographic analysis consisted in determining the content of aucubin and catalpol in the test samples by applying the external standard method based on calibration curves generated by a validated HPLC method. Each test sample was subjected to a series of three measurements, and the arithmetic mean and standard deviation were determined from the measurement results.

3. Differential Scanning Calorimetry (DSC)

Before starting the measurements, the equipment was calibrated, and a 40 µL aluminum pan was filled with a lipid sample or a sample of the dispersion of lipid nanoparticles under study. The measurement principle was to gradually heat the sample from 25 °C to 90 °C in nitrogen flow (20 mL/min) at a scanning rate of 5 °C per minute, keep the sample at 90 °C for 1 min, and then cool it down to 25 °C at similar parameters.

4. Release Study of Iridoid Glycosides from Cosmetic Formulations

4.1. Materials

Lipid phase components—Creagel EZ® 7, Alphaflow® 20 (Créations Couleurs, Dreux, France) and a preservative—Microcare® SB (a mixture of sodium benzoate and

potassium sorbate; Thor GmbH, Erfurt, Germany) were used to prepare the O/W emulsions. Potassium dihydrogen phosphate and sodium hydroxide (Merck, Darmstadt, Germany) were used to perform the release study.

4.2. Preparation Of Cosmetic Formulation (O/W Emulsion) Enriched with Lipid Nanoparticles

The Creagel EZ® 7 (9.8 wt.%) and Alphaflow® 20 (16.7 wt.%) were weighed into a glass beaker and thoroughly mixed. Then, the lipid nanoparticles dispersion obtained earlier and Microcare® SB (0.2 wt.%) were gradually added upon vigorous stirring the resulting emulsion until the desired consistency was obtained.

4.3. Release Study—Methodology

A study of the release efficiency of iridoid glycosides from O/W emulsions was carried out using a 708-DS Dissolution Apparatus (Agilent Technologies, Santa Clara, CA, USA) in combination with a high-performance liquid chromatograph Varian 920-LC. The phosphate buffer (pH=5.8) was used as a medium/acceptor fluid and Cuprophan as a membrane imitating the epidermis of the skin. The analysis was conducted for 24 h and the measurements were performed every 30 min until the 6th hour of the release study and then at hourly intervals. The subsequent chromatographic analysis was performed on the samples of 1 mL of acceptor fluid from a glass vessel from above the extraction chamber containing the tested cosmetic emulsion. The percentage value of released iridoid glycosides were calculated on the basis of their concentration in acceptor fluid and comparison to the gross content in the cosmetic sample.

4.4. Release study—results

To evaluate the efficiency of release of iridoid glycosides from the cosmetic preparations, they were allowed to penetrate from the O/W emulsion to the acceptor fluid, where the subsequent changes in the concentration of aucubin and catalpol were determined using the high-performance liquid chromatography. The percentage of released aucubin and catalpol from O/W emulsion as a function of time is presented in the figure below.

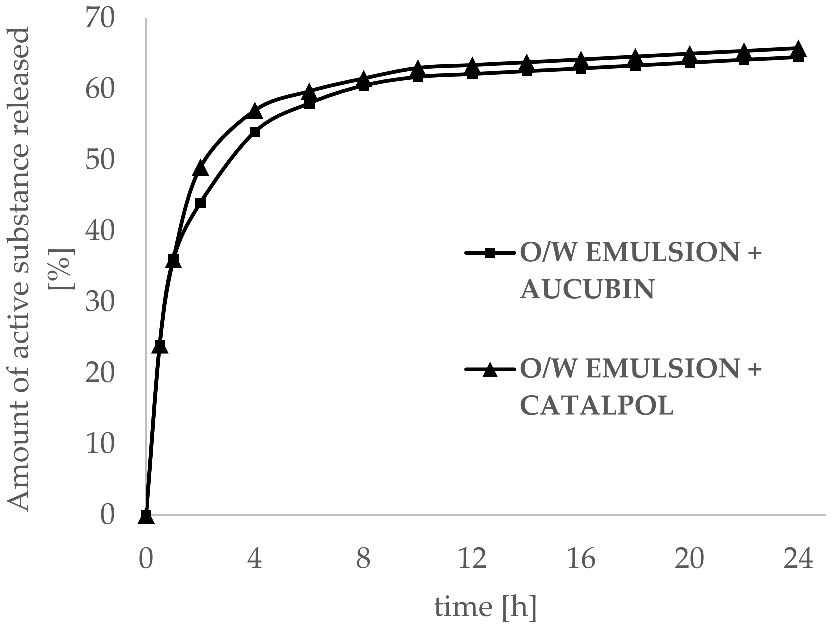

Figure S1. Efficiency of release of iridoid glycosides from the cosmetic preparations.

No significant differences in the release efficiency of aucubin and catalpol from the prepared cosmetic products were observed; their release reached 64.5% and 65.8%, respectively. Moreover, no characteristic biphasic release profile was observed—iridoid glycosides were released from the emulsion gradually and the shape of the release profiles was determined as classical, without the effect of rapid release of the active substance. The differences in the type of release are attributed to the method used to obtain lipid nanoparticles (based on a multiple emulsion), during which the incorporation of active ingredients occurs into the aqueous phase of the internal emulsion. The obtained results correspond to those reported by other scientists, e.g. Farboud (Farboud et al.) [31] who in the course of research on the efficiency of coenzyme Q10 release (incorporated into SLNs) from O/W cream encountered an analogous way of releasing the active substance. In our previous work, we also carried out studies on the release efficiency of non-encapsulated glycosides from selected cosmetic formulations—the obtained release rates were on average 5–10% lower when compared to those in the cosmetics containing aucubin and catalpol in the form of lipid nanoparticles.

Table S1. DSC thermal analysis–parameters determined for the lipid and the studied dispersions of lipid nanoparticles**.**

	**Melting Point [°C]**	**Enthalpy [J/g]**
SOFTISAN® 100	42.73	−47.36
NON-INCORPORATED LIPID NANOPARTICLES	38.96	−1.64
AUCUBIN-LOADED LIPID NANOPARTICLES	35.12	−0.79
CATALPOL-LOADED LIPID NANOPARTICLES	34.89	−0.83
